# Widespread temporo-occipital lobe dysfunction in amyotrophic lateral sclerosis

**DOI:** 10.1038/srep40252

**Published:** 2017-01-09

**Authors:** Kristian Loewe, Judith Machts, Jörn Kaufmann, Susanne Petri, Hans-Jochen Heinze, Christian Borgelt, Joseph Allen Harris, Stefan Vielhaber, Mircea Ariel Schoenfeld

**Affiliations:** 1 Department of Neurology, Otto-von-Guericke University, Leipziger Straße 44, 39120 Magdeburg, Germany; 2 Department of Computer Science, Otto-von-Guericke University, Universitätsplatz 2, 39106 Magdeburg, Germany; 3 German Center for Neurodegenerative Diseases (DZNE), Leipziger Straße 44, 39120 Magdeburg, Germany; 4 Department of Neurology, Hannover Medical School, Carl-Neuberg-Str. 1, 30625 Hannover, Germany; 5 Leibniz Institute for Neurobiology, Brenneckestraße 6, 39118 Magdeburg, Germany; 6 Kliniken Schmieder Heidelberg, Speyererhofweg 1, 69117 Heidelberg, Germany

## Abstract

Recent studies suggest that amyotrophic lateral sclerosis (ALS) and frontotemporal dementia (FTD) lie on a single clinical continuum. However, previous neuroimaging studies have found only limited involvement of temporal lobe regions in ALS. To better delineate possible temporal lobe involvement in ALS, the present study aimed to examine changes in functional connectivity across the whole brain, particularly with regard to extra-motor regions, in a group of 64 non-demented ALS patients and 38 healthy controls. To assess between-group differences in connectivity, we computed edge-level statistics across subject-specific graphs derived from resting-state functional MRI data. In addition to expected ALS-related decreases in functional connectivity in motor-related areas, we observed extensive changes in connectivity across the temporo-occipital cortex. Although ALS patients with comorbid FTD were deliberately excluded from this study, the pattern of connectivity alterations closely resembles patterns of cerebral degeneration typically seen in FTD. This evidence for subclinical temporal dysfunction supports the idea of a common pathology in ALS and FTD.

Amyotrophic lateral sclerosis (ALS) is a neurodegenerative disease characterized by the progressive loss of both upper and lower motor neurons with a mean survival of 2–3 years from symptom onset^1^. Although the degeneration of the motor system is the primary hallmark of the disease, varying degrees of extra-motor symptoms are also observed. For example, up to 50% of ALS patients show neuropsychological deficits^2^, with 12–15% fulfilling the criteria for comorbid frontotemporal dementia (FTD)^3^,^4^. The discovery of the GGGGCC hexanucleotide expansion on chromosome 9 (*C9orf72*)^5^,^6^ in families with cases of ALS, FTD, and ALS-FTD established a genetic link between the two conditions and supports previous observations of common neuropathological abnormalities in ALS and FTD (i.e., the accumulation of the DNA/RNA binding protein TDP-43)^7^. These findings have led to the idea of a single disease continuum, the ends of which are represented by ALS and FTD, and shifted the focus towards ALS-related extra-motor changes.

Despite the clinical, genetic, and histopathological overlap between the two diseases, results from functional neuroimaging studies were rather inconclusive in this regard. In particular, it seems surprising that only limited involvement of temporal lobe regions was observed in ALS patients, although these regions are typically affected in frontotemporal neurodegeneration^8^.

To investigate this observation at the network level, we examined intrinsic functional connectivity differences between ALS patients and healthy controls. Importantly, ALS-FTD patients were deliberately excluded to ensure that changes did not result from comorbid FTD. Whole-brain graph analysis was carried out at voxel resolution, thus enabling more detailed insights into the patterns of ALS-related network changes through enhanced spatial sensitivity. We expected to find neural correlates of subclinical changes in extra-motor areas, including the temporal lobes.

## Results

### Neuropsychology

Neuropsychological deficits in ALS patients were limited to the domains of executive function and verbal memory. More specifically, performance deficits were observed in letter fluency and flexibility, Stroop test ratio and error rates, trail making test ratio, and recognition memory. There were no between-group differences in semantic processing, visuo-spatial skills, or verbal memory encoding and recall. With respect to behavioral changes, patients showed higher levels of apathy than controls. No changes were detected for the Frontal Systems Behavioral Scale (FrSBe) subscales corresponding to disinhibition and executive dysfunction. The detailed results from neuropsychological testing are presented in Table 1.

### Functional connectivity

**Figure 1:**
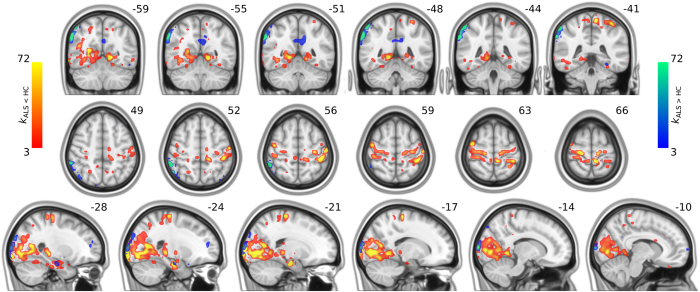
Between-group differences in connectivity: degree maps. Degree maps *k*_*ALS < HC*_ and *k*_*ALS > HC*_ are superimposed on top of MNI slices in order to map clusters of decreased and increased connectivity in ALS, respectively. The degree *k*_*ALS < HC*_ (*k*_*ALS > HC*_) of a voxel/node *v* is the number of pairs *(v, w)* in *G*_*t*_, exhibiting significantly decreased (increased) functional connectivity (FDR < 0.05), where *w* can be any other voxel than *v*.

The whole-brain voxel-level graph analysis revealed complex patterns of both decreased and increased connectivity in ALS patients as compared to controls (Fig. 1). Specifically, clusters of reduced functional connectivity were observed in cortical sensorimotor (bilateral pre- and postcentral gyrus), parietal (left superior parietal, right angular gyrus), temporal (bilateral temporal pole, left planum temporale, bilateral superior and inferior temporal gyrus, right middle temporal gyrus, bilateral parahippocampal gyrus and fusiform cortex), occipital (bilateral fusiform cortex, lateral occipital cortex, cuneal cortex, lingual gyrus, occipital pole and intracalcarine cortex, right precuneus, right supracalcarine cortex), frontal (bilateral frontal pole, left insular cortex, right middle frontal gyrus, right operculum cortex), and subcortical regions (bilateral thalamic nuclei, left amygdala, bilateral hippocampi, bilateral caudate nuclei, left nucleus accumbens).

**Figure 2:**
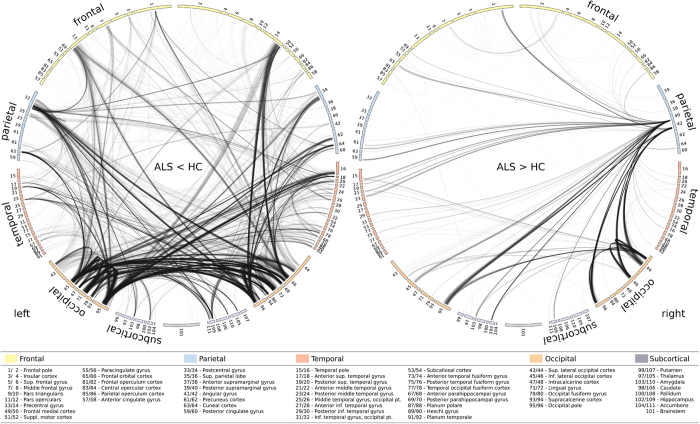
Between-group differences in connectivity: connectograms. ALS-related functional connectivity changes are illustrated at the level of individual voxel pairs using connectograms. Each circular segment corresponds to a region from the Harvard-Oxford atlas. Each voxel was assigned a unique position on the segment of its comprising region using multidimensional scaling based on the pairwise similarity of the voxels’ connectivity profiles with respect to *G*_*t*_. A link connecting two voxels indicates significantly decreased (left) or increased (right) functional connectivity in ALS (FDR < 0.05).

Focusing on the motor system, patients with ALS showed impaired long-range functional connectivity between primary motor and sensorimotor regions (pre- and postcentral gyrus) and the occipital pole, affecting both intra- and interhemispheric connections (Fig. 2). Along with the motor system, the temporo-occipital cortex exhibited extensive connectivity changes. Specifically, ALS-related connectivity decreases were observed between temporal and occipital lobe areas incorporating short-range connections, as well as those connected across cerebral hemispheres. Functional connectivity was also decreased between cortico-subcortical areas, involving connections among bilateral hippocampi and occipital lobe areas, as well as the right thalamus and left temporal lobe areas.

The analysis also revealed clusters of increased connectivity associated with ALS (Fig. 1), located in cortical sensorimotor (right pre- and postcentral gyrus), frontal (left inferior frontal gyrus, left insular cortex, bilateral frontal pole), temporal (left temporal pole, left planum polare, right fusiform gyrus), occipital (bilateral lateral occipital cortex, left precuneus), parietal (right angular gyrus), and subcortical regions (right hippocampus), though these connectivity increases were somewhat less pronounced than the observed decreases. Further connectivity increases were observed between the right parietal lobe and the left temporal pole, as well as between the right occipital lobe and the frontal lobes. These clusters resulted in large parts from increased interhemispheric connectivity (Fig. 2).

**Figure 3:**
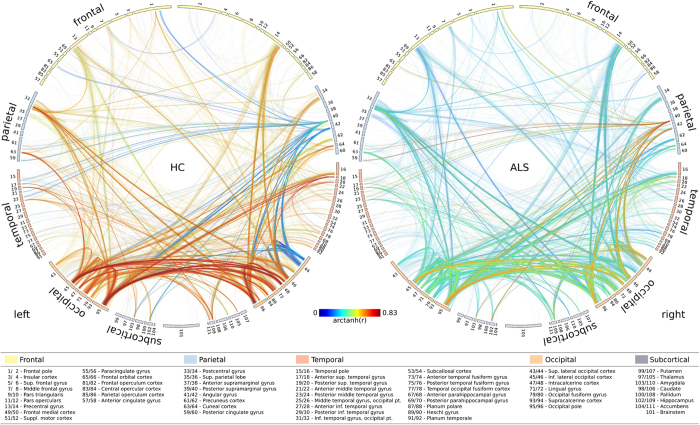
Group-specific averages of connectivity. Connectograms display the group-specific average connectivity of the significant connections for patients with ALS (left) and healthy controls (right).

Figure 3 illustrates the group-specific average connectivity for each voxel-pair in healthy controls and patients with ALS, respectively. Notably, Fisher z-transformed correlation values ranged from 0 to 0.83 (corresponding to the quantiles *Q*_*0.01*_ and *Q*_*0.99*_, respectively) for both groups. No negative correlations were observed. Cohen’s *d* effect sizes, computed for each pair of nodes exhibiting a significant between-group difference in connectivity, were greater than 0.8.

## Discussion

The present study investigated the neural correlates of degeneration in ALS patients. Using functional MRI at rest in conjunction with a whole-brain voxel-level approach, we examined functional connectivity alterations in ALS patients. Consistent with the hallmark pathology of the disease, we observed prominent clusters of decreased functional connectivity in motor-related areas (Fig. 1), which were predominantly characterized by many affected long-range connections (Fig. 2). Strikingly, the analysis also revealed widespread patterns of decreased functional connectivity along the temporal and occipital lobes (Fig. 1), a pattern resembling the neurodegenerative changes in FTD patients. This is of the utmost importance since ALS-FTD patients were explicitly excluded from the study, and our ALS patients exhibited only minor cognitive deficits, especially with regard to temporal lobe dysfunction. The current results provide *in vivo* evidence for the involvement of the temporal lobe in non-demented ALS patients, supporting recent genetic^5^,^6^, and histopathological^7^ reports pointing to a shared pathology between ALS and FTD.

The present results add to the body of literature providing a comprehensive account of the neural connectivity changes accompanying the progression of ALS. A previous study from our group employed structural and functional MRI in conjunction with a longitudinal design and reported a drop in functional activity in the motor cortex reflecting the breakdown of compensatory mechanisms within only 3 months of a diagnosis^9^. This study also reported increased hippocampal activity across the same time range, presumably reflecting a mechanism of compensation^9^. A second study employed diffusion-weighted MRI and found decreased structural connectivity in the motor system but increased diffusivity along occipito-temporal pathways^10^. In the present study, we report prominent clusters of reduced functional connectivity in the motor cortex, completing the picture of pathological changes over the course of the disease. It would appear then, that ALS starts with subclinical neurodegenerative changes in the motor cortex, for which, at least in the very early stages, higher activity in non-affected motor neurons compensates^11^. Shortly after the first clinical symptoms appear, the neurodegeneration can no longer be compensated for, and a decay of upper and lower motor neuron function follows^12^, resulting in decreased BOLD activity in the motor cortex^9^, as well as diffusivity along the pyramidal pathways^10^.

The observed connectivity reductions in the motor cortex of ALS patients were primarily driven by affected long-range connections (occipital and temporal) to and from the motor cortex, and are in line with previously shown structural and functional changes in the motor system^13^,^14^. Functionally, the connectivity between motor areas and the visual cortex plays a key role in integrating visuo-motor information through cortico-cortical links^15^ and its observed degradation is likely a consequence of neuronal cell loss in primary motor regions. In sum, there is converging evidence from clinical and neurophysiological measures on the neurodegeneration and its temporal dynamics within the motor system. In terms of the temporal dynamics of disease progression, structural connectivity appears sensitive to alterations occurring over a longer time, whereas BOLD-related measures such as fMRI or measures of functional connectivity provide snapshots of ongoing dynamic processes evolving over shorter time periods. Therefore, depending on the time point of measurement, they can show either increased (in the early subclinical stages) or decreased (in the more advanced clinical stages) values.

Since the typically studied ALS patient population is heterogeneous, and includes patients with comorbid frontotemporal dementia, the involvement of the temporal lobe as an integral component of the disease is less clear. As suggested by recent work^11^,^16^, we excluded patients with ALS-FTD from the current study in order to address this issue. Importantly, we observed striking patterns of decreased functional connectivity across the occipital and temporal lobes. The extent of these patterns was surprising, especially given that the included patients exhibited only minor cognitive deficits, as well as the fact that all ALS-FTD patients were excluded. The connectivity decreases in the temporal lobe closely resemble the pattern of previously described functional network degeneration in patients with FTD^8^ and speak in favor of a clear involvement of the temporal and occipital lobes in ALS, even in cognitively unimpaired patients. In light of common clinical^3^, genetic^5^,^6^, and histopathological^7^ characteristics shared by ALS and FTD, these observations argue in favor of a single continuum upon which both conditions might lie.

Importantly, similar patterns of connectivity reductions have also been observed in pre-symptomatic familial FTD^17^, suggesting that distinctive functional connectivity alterations might emerge before the first cognitive symptoms appear. A long pre-symptomatic period typically precedes the clinical phase of neurodegenerative disorders such as Parkinson’s Disease^18^ or Alzheimer’s Disease^19^, and has also recently been proposed for ALS^20^. In contrast to MRI-based measures of structural connectivity^10^, functional measures relying on the BOLD effect in the same data set were sensitive enough to detect small changes^9^ and therefore to delineate the dynamics of pathology-related transient changes of neural activity in ALS. Here, our whole-brain connectivity approach detected ALS-related changes in the temporal lobe in a presumably pre-symptomatic phase, strongly suggesting that these changes reflect disturbances in neuronal homeostasis and functioning before cell-loss. In this context, connectivity-based biomarkers could facilitate earlier, perhaps even pre-symptomatic diagnosis and treatment. In summary, the results presented here – widespread functional connectivity reductions in the temporal and occipital lobes in non-demented ALS patients – constitute compelling *in vivo* evidence for a shared pathology with FTD and pre-symptomatic connectivity changes in the temporal lobe.

In addition, the observed connectivity decreases also included large areas of the occipital cortex, characterized by impaired long-range connections with ipsi- and contralateral motor areas, interhemispheric occipital connections, as well as short-range connections with adjacent areas of the occipital and temporal cortex. ALS-related alterations within primary and associative visual areas have been observed before, including gray matter atrophy^21^, cortical thinning^22^, decreased glucose metabolism^23^,^24^, and reduced functional connectivity^25^, but were never related to clinical impairment. Since none of our patients reported overt visual perception deficits, it is reasonable to assume that the observed connectivity reductions in the present study most likely result from disrupted connections within the temporo-occipital lobe along the ventral pathway^15^ (Fig. 2). Whether these changes might be related to subtle deficits in visual perception needs to be investigated in further studies focusing on this question.

The analysis also revealed distinct, albeit relatively sparse, patterns of increased functional connectivity in the frontal and parietal lobes. These areas were recently reported to be part of an expanding disruption of structural connectivity spreading from primary motor areas to frontal and parietal regions over the course of 5.5 months^26^. The observed functional connectivity increases could reflect regulatory processes that might serve to overcome the beginning structural lesion in the frontal and parietal lobes. These areas are also known to be involved in deficits in executive function and visuo-spatial processing frequently encountered in ALS^27^. In fact, the relatively minor neuropsychological executive deficits exhibited by the patients in the present study could indeed be a consequence of successful functional compensation. In support of this notion, we observed increased connectivity within the frontal, parietal, temporal, and occipital lobes (Fig. 1b). However, we also found increased interhemispheric connectivity, particularly between the parietal, temporal, and occipital lobes, possibly reflecting a loss of inhibitory GABAergic cortical interneurons^28^,^29^ as a result of corpus callosum damage consistently reported in ALS^16^,^30^. In summary, the observed patterns of increased connectivity seem better explained by a combination of both compensation and transcallosal disinhibition rather than by one of these mechanisms alone.

In combination, these findings – decreased structural^13^ and functional connectivity in primary motor areas, increased functional connectivity in the frontal and parietal lobes, and decreased functional connectivity in the temporo-occipital lobe – suggest distinct ALS stages for the involvement of different functional networks. Motor network degeneration seems to proceed primarily in earlier, partly presymptomatic phases, for which concomitant increases in motor activity in unaffected areas compensate^11^. With disease progression, these compensatory mechanisms break down^9^, which is in line with the observed reductions in motor functional connectivity. In contrast, adjacent extra-motor areas in the frontal and parietal lobes are affected at later stages of the disease, and increases in functional connectivity can be interpreted as a mixture of compensatory mechanisms counteracting the evolving structural lesion^11^ and transcallosal disinhibition^16^. Finally, the temporo-occipital cortex is targeted relatively late in the neurodegenerative process^31^,^32^, and although it is not yet affected by extensive cell loss, it already exhibits marked reductions in functional connectivity. It seems conceivable that these connectivity reductions emerge before the damage caused by progressive neurodegeneration becomes observable through structural imaging methods.

There are some limitations to consider in this study. The described edge-level significance assessment, controlling the false discovery rate (FDR), does not exploit any spatial or topological information, which limits its sensitivity. Different methods, controlling for family-wise errors (FWE) at the level of connected components (NBS) or spatial pairwise clusters (SPC) based on permutations, would be preferable in this regard^33^,^34^. However, because of the computational burden associated with the permutations, these approaches are currently impractical to apply to voxel-level graphs. Furthermore, these methods provide statistical assessment at the level of components or clusters, so that the individual connections comprising such components or clusters cannot be declared significant^34^.

The included patient cohort only exhibited minor cognitive deficits with some of the patients showing higher apathy levels as measured with the FrSBe. Recent studies suggest that apathy levels in ALS should rather be assessed by specifically designed apathy scales since the FrSBe does not account for the patients’ motor impairment and reports a high number of false positive results with regard to apathy symptoms^35^. However, none of these patients fulfilled the criteria for behavioral variant frontotemporal dementia, and disinhibition and executive dysfunction scores were not different from those observed in the healthy control group, suggesting that it is rather unlikely that apathy drives the temporal lobe connectivity reductions.

In conclusion, we investigated functional connectivity patterns across the whole brain of ALS patients and compared these to healthy controls using functional MRI. In addition to motor dysfunction, the employed graph-based analysis at high spatial resolution revealed widespread temporal lobe involvement at the neurophysiological level although no clinical signs of dysfunction were evident at the behavioral level. This evidence suggests subclinical temporal dysfunction to be a core component of ALS and provides support for a common pathology in ALS and FTD. Connectivity analyses of functional MRI data at high spatial resolution have the potential to detect dysfunctions at subclinical stages and are very promising for the development of biomarkers for neurodegenerative diseases, in which substantial degenerative changes occur before the first clinical signs appear.

## Methods

### Participants

We studied 64 patients with ALS and 38 healthy controls. Patients were classified according to the revised El Escorial Criteria^36^, and clinical severity was rated using the revised ALS Functional Rating Scale (ALSFRS-R)^37^. Patients were recruited from the outpatient clinics of the Hannover Medical School (Hannover, Germany) and the Otto-von-Guericke University (Magdeburg, Germany). To maximize clinical homogeneity, patients with progressive muscular atrophy, primary lateral sclerosis, or concomitant FTD were not included in this study. FTD diagnoses were made based on current diagnostic criteria^38^,^39^ and included the interview of the caregiver. Exclusion criteria for all participants included cerebrovascular disease, traumatic brain injury, and other neurologic or psychiatric diseases. The controls were matched to the patient group for age, sex, and education. Moreover, the controls were screened for cognitive impairment and were included only if they scored within the normal range of the Montreal Cognitive Assessment (MoCA). The study was approved by the ethics committee of the Otto-von-Guericke University (Reference number: 75/11), and was carried out in accordance with the relevant guidelines and regulations. All participants gave written informed consent prior to inclusion. Demographic data are summarized in Table 2.

### Neuropsychological assessment

All participants underwent detailed neuropsychological assessment to test executive, memory, language, and visuo-spatial functions. Executive function was tested using the Trail Making Test (TMT), a computerized version of the Stroop Test, the backward digit span task from the revised Wechsler Memory Scale (WMS-R), and the Regensburger Verbal Fluency Test (RWT). To account for motor impairment, fluency and flexibility indices were computed as suggested by Abrahams and colleagues^40^. To assess memory function, the forward digit span task and the German version of the Rey Auditory Verbal Learning Test (VLMT) were employed. Semantic language function was assessed using the subtests 2 (semantic categorization according to main features) and 3 (semantic categorization according to sub-features) from the Bogenhausen Semantic Test (BOSU), and visuo-spatial skills were tested using the copy subtest of the Rey Complex Figure Test (RCFT). Tests were adapted to speech and motor deficits by analyzing tempo-independent (TMT and Stroop ratio) and tempo-adapted (fluency indices) scores. Nevertheless, due to the range of physical disabilities in the ALS cohort, not all patients were able to complete all tests (Table 1). These patients were excluded from the individual analyses while retaining them in the data set. Behavioral data were available for a subset of patients (n = 41) and controls (n = 22) for apathy, disinhibition, and executive dysfunction using the Frontal Systems Behavioral Scale (FrSBe).

The distributions of demographic and neuropsychological variables were tested for normality using the Kolmogorov-Smirnov test. Differences in education, gender, and handedness were assessed using chi-square (gender, handedness) and Mann-Whitney *U* (education) tests. Age was normally distributed and between-group differences were tested using an independent two-sample *t* test. Neuropsychological test variables were not normally distributed, and between-group differences were assessed using the non-parametric Mann-Whitney *U* test (*α* = 0.05).

To assess the degree of impairment, cut-off values for each individual test were calculated based on the performance of the healthy controls. A patient was considered impaired with regard to a given test if the performance was more than two standard deviations below the mean of the healthy controls.

### MRI data acquisition

All imaging data were acquired on a Siemens Magnetom Verio 3 T MRI scanner (Siemens, Erlangen/Germany) with a 32-channel head coil. A high-resolution, T1-weighted structural scan was obtained for anatomical reference using a 3D-MPRAGE sequence (TE = 4.82 ms, TR = 2500 ms, TI = 1100 ms, flip angle = 7°, voxel size = 1 mm^3^). Resting-state fMRI scans were acquired using an echo-planar imaging (EPI) sequence (TE = 30 ms, TR = 2200 ms, flip angle = 80°, voxel size = 3.5 × 3.5 × 3.5 mm^3^) sensitive to blood oxygen level-dependent (BOLD) contrast. A total of 302 volumes per subject were acquired with the first two volumes automatically being discarded by the scanner. Slices were obtained parallel to the intercommissural (AC-PC) line. Participants were instructed to close their eyes and remain awake during acquisition. To address the problem of geometric distortions in EPI caused by magnetic field inhomogeneity, a B0 field map was acquired prior to the EPI sequence using a double-echo gradient recall echo (GRE) sequence (TE 1/2 = 4.92 ms/7.38 ms, TR = 675 ms, flip angle = 60°, voxel size = 2.6 × 2.6 × 2.0 mm^3^).

### MRI data preprocessing

For each subject, structural images were skull-stripped^41^ and warped to MNI space using FLIRT^42^ and FNIRT^43^ (T1-to-MNI registration). Functional images were corrected for time differences in slice acquisition (slice time correction), despiked using AFNI’s 3dDespike, and realigned to the mean functional image using 6-DOF rigid body transformations to compensate for head motion^42^ (motion correction). Geometric distortions induced by magnetic field inhomogeneity were corrected based on the GRE field map (EPI distortion correction) and the data were registered to the corresponding structural scan (EPI-to-T1 registration). EPI distortion correction and EPI-to-T1 registration were performed simultaneously using FSL’s epi_reg. The spatial transformations from motion correction, EPI distortion correction, EPI-to-T1 registration, and T1-to-MNI registration were combined into a single warp to reduce interpolation-induced blurring^44^. Using this warp, the slice time corrected and de-spiked functional data were transformed from native to standard space in one resampling step. The resulting images were spatially smoothed using a Gaussian kernel (7 mm FWHM) to improve the signal-to-noise ratio and to further accommodate inter-individual anatomic variations. Confound regression was performed using 12 parameters (the 6 parameters from the motion correction step and their temporal derivatives). To account for low frequency intensity drifts and high frequency noise, the data were bandpass-filtered (0.01–0.1 Hz).

### Functional connectivity analysis

Subject-specific connectivity graphs were constructed by defining gray matter voxels as nodes and establishing weighted edges by estimating internodal functional connectivity in terms of Pearson correlation between the nodes’ associated time series. After applying the Fisher z-transformation to the correlation values, edge-level *t* statistics were computed across graphs to assess between-group differences in connectivity. To be able to compute these statistics in a memory-efficient fashion, we used on-demand connectivity estimation^45^. The resulting graph of statistics *G*_*t*_was used to derive the graph of *p* values *G*_*p*_^46^. *G*_*p*_ was then pruned based on *q* value estimation^47^ in order to identify edges (i.e., pairs of nodes/voxels) that exhibit significant differences (FDR < 0.05). Based on their direction^45^, the so-obtained graph *G*_*s*_ formed by the remaining edges, was partitioned into the two subgraphs *G*_*s<*_ and *G*_*s>*_ corresponding to ALS-related decreases and increases in connectivity, respectively. Edge-level effect sizes were calculated using Cohen’s *d*.

To visualize *G*_*s<*_ and *G*_*s>*_, we used circos^47^ to generate connectograms^49^ that were specially designed to accommodate the illustration of voxel-level information. Specifically, each circular segment in a voxel-level connectogram corresponds to a region of the Harvard-Oxford atlas^50^, and each voxel is assigned a unique position on the segment of its comprising region using multidimensional scaling based on the pairwise similarity of the voxels’ connectivity profiles with respect to *G*_*t*_. A link connecting two voxels indicates significantly decreased or increased functional connectivity in ALS as compared to controls for these voxels. To display the group-specific average connectivity of the significant connections, separate connectograms were generated for the ALS patients and healthy controls, respectively.

## Additional Information

**How to cite this article**: Loewe, K. *et al*. Widespread temporo-occipital lobe dysfunction in amyotrophic lateral sclerosis. *Sci. Rep.* **7**, 40252; doi: 10.1038/srep40252 (2017).

**Publisher's note:** Springer Nature remains neutral with regard to jurisdictional claims in published maps and institutional affiliations.

## Figures and Tables

**Table 1:** Neuropsychological performance.

Cognitive Measure	Group	N	Mean	SD	Cut-off	No. of impaired patients	*U*	*p*
**Memory**								
VLMT Learning (∑1-5)	HC	38	50.71	8.4	33.91	4 (7.3%)	935.0	0.390
	ALS	55	48.78	10.7				
VLMT Immediate Recall (6)	HC	38	10.68	2.8	5.08	6 (10.9%)	918.5	0.320
	ALS	55	9.91	3.4				
VLMT Delayed Recall (7)	HC	38	10.55	3.0	4.55	4 (7.3%)	926.0	0.349
	ALS	55	9.85	3.4				
VLMT Recognition (corrected for errors)	HC	38	12.50	2.8	6.90	9 (16.4%)	767.0	0.028
	ALS	55	10.62	4.5				
WMS-R Digit span forward	HC	38	7.45	1.6	4.25	2 (3.3%)	1090.0	0.710
	ALS	60	7.35	1.8				
**Executive function**								
RWT Letter fluency index	HC	37	3.91	1.4	6.71	8 (15.1%)	757.5	0.067
	ALS	53	4.82	2.3				
RWT Letter flexibility index	HC	37	4.34	1.5	7.34	10 (19.6%)	706.0	0.045
	ALS	51	5.96	4.4				
RWT Semantic fluency index	HC	37	2.03	0.7	3.43	3 (5.9%)	789.0	0.192
	ALS	51	2.32	1.0				
RWT Semantic flexibility index	HC	37	3.35	0.9	5.15	5 (9.8%)	759.0	0.119
	ALS	51	3.87	1.7				
Stroop ratio	HC	37	1.06	0.2	1.46	3 (6.1%)	577.0	0.004
	ALS	49	1.20	0.3				
Stroop errors	HC	37	0.54	1.0	2.54	11 (22.5%)	687.5	0.030
	ALS	49	2.10	4.2				
TMT Cognitive flexibility	HC	38	2.26	0.8	3.86	6 (11.8%)	629.5	0.022
	ALS	51	2.72	1.2				
WMS-R Digit span backward	HC	38	6.32	1.6	3.12	3 (5.0%)	893.5	0.065
	ALS	60	5.70	1.6				
**Language**								
Semantic Categorization								
BOSU Main features	HC	38	9.87	0.4	9.07	6 (9.8%)	1148.0	0.880
	ALS	61	9.90	0.3				
BOSU Sub features	HC	38	9.16	1.0	7.16	4 (6.6%)	1124.0	0.787
	ALS	61	9.13	0.9				
**Visuospatial skills**								
RCFT (copy)	HC	27	32.80	2.5	27.80	4 (11.1%)	380.5	0.139
	ALS	36	31.47	3.5				
**Behaviour**								
FrSBe Apathy	HC	22	22.36	5.1	32.56	15 (36.6%)	260.0	0.006
	ALS	41	29.0	9.5				
FrSBe Disinhibition	HC	22	22.36	5.4	33.16	3 (7.3%)	336.0	0.097
	ALS	41	24.8	5.8				
FrSBe Executive Dysfunction	HC	22	30.0	10.6	51.20	1 (2.4%)	356.5	0.173
	ALS	41	32.4	8.9				

VLMT: Rey Auditory Verbal Learning Test; WMS-R: Wechsler Memory Scale revised; RWT: Regensburger Verbal Fluency Test; TMT: Trail Making Test; BOSU: Bogenhausen Semantic Test; RCFT: Rey Complex Figure Test; FrSBe: Frontal System Behavioral Scale.

**Table 2:** Demographic profile of participants.

Group	No.	Age [years]	Sex [male]	Handedness [right]	Education [years]	limb -bulbar onset	ALSFRS-R	Disease duration [months]
HC	38	59.5 ± 10.2	26	38	11.1 ± 1.0	n.a.	n.a.	n.a.
ALS	64	58.9 ± 11.5	39	59	10.6 ± 1.1	46−18	38.9 ± 5.7	20.4 ± 15.0
*p*	—	0.77	0.45	0.08	0.06	—	—	—
